# Increased climate pressure on the agricultural frontier in the Eastern Amazonia–Cerrado transition zone

**DOI:** 10.1038/s41598-021-04241-4

**Published:** 2022-01-10

**Authors:** José A. Marengo, Juan C. Jimenez, Jhan-Carlo Espinoza, Ana Paula Cunha, Luiz E. O. Aragão

**Affiliations:** 1 CEMADEN, São Jose dos Campos, SP Brazil; 2 GCU/IPL, Univerdity of Valencia, C/Catedratico Jose Beltran, 46980 Paterna, Valencia Spain; 3 Université Grenoble Alpes, IRD, CNRS, G-INP, IGE (UMR 5001), Grenoble, France; 4 Remote Sensing Division, National Institute for Space Research INPE, Av. dos Astronautas, 1.758, 12227-010 Sao José dos Campos, Brazil

**Keywords:** Climate sciences, Environmental sciences

## Abstract

Several large-scale drivers of both anthropogenic and natural environmental changes are interacting nonlinearly in the transition zone between eastern Amazonia and the adjacent Cerrado, considered to be another Brazilian agricultural frontier. Land-use change for agrobusiness expansion together with climate change in the transition zone between eastern Amazonia and the adjacent Cerrado may have induced a worsening of severe drought conditions over the last decade. Here we show that the largest warming and drying trends over tropical South America during the last four decades are observed to be precisely in the eastern Amazonia–Cerrado transition region, where they induce delayed wet-season and worsen severe drought conditions over the last decade. Our results evidence an increase in temperature, vapor pressure deficit, subsidence, dry-day frequency, and a decrease in precipitation, humidity, and evaporation, plus a delay in the onset of the wet season, inducing a higher risk of fire during the dry-to-wet transition season. These findings provide observational evidence of the increasing climatic pressure in this area, which is sensitive for global food security, and the need to reconcile agricultural expansion and protection of natural tropical biomes.

## Introduction

Land-use change for agrobusiness expansion together with underlying climate change may induce higher frequency of extreme climate events^1–6^, increasing the exposure and vulnerability of tropical forests and Cerrado^7–9^. The transition zone between the Eastern Amazon and the Cerrado (EAC) biomes comprises the largest area of contact between forest and savanna in the tropics, with the Cerrado recognized as the world’s most biodiverse savanna^10^. The hypothesis of “savannization” of Amazonia suggests that such a new equilibrium state becomes more likely as the climate gets warmer and drier, deforestation advances and fires become more frequent^11^. The expected result of this interplay of processes is a contraction of the humid and dense forests giving way to a Cerrado-like biome. Modeling studies show that the Amazon may have “tipping points”^8^ linked to their exceeding of deforestation and temperature thresholds^12^. Satellite-based observations have recently revealed that the area of degradation and natural disturbance tis surpassing that impacted by deforestation in the Amazon region^3,4^. Acting synergistically with processes already in play in the Amazon, the deterioration described here may increase climate change pressure in the region, especially putting at risk productive areas responsible for supporting global food security^5,6^.

In the EAC, the MATOPIBA region (which includes the states of Maranhão, Tocantins, Piauí and Bahia—Extended Data Fig. 1) became an important agricultural frontier during the past 20 years^1^. The Cerrado is the dominant biome in the MATOPIBA (91% of the area), which also has patches of Amazon Forest and the Caatinga vegetation (shrubland vegetation typical of northeastern Brazil). The Cerrado biome is the second largest biome in Brazil, only 0.85% of its area is legally protected, and it is the biome with the most important area in terms of grain production in Brazil^13^.

The current economic scenario continues to conspire against the Amazon by placing a higher premium on agricultural commodities such as soybeans, meat, and tropical timber than on standing forests^7^. The agricultural development in the MATOPIBA region in the EAC is an example of this. To prioritize deforestation-free agricultural expansion here, it is critical to increase pasture productivity coupled with incentives for direct agricultural expansion over already-converted lands^14^. In the last decade, more than half of the agricultural expansion in the Cerrado occurred over pasture areas, except for the MATOPIBA region which had the greatest expansion over native vegetation areas because of the lack of already-disturbed areas suitable for agriculture^15^.

In the EAC, rainfall seasonality, dryness, higher temperatures, and fire disturbances have important ecological impacts, much to the detriment of biodiversity, water availability, and traditional peoples’ livelihoods and lives. These changes may affect the future of the Amazon and Cerrado biomes, which already are more susceptible to drought^2^. The total amount of precipitation received over EAC varies from about 500 mm year^−1^ in the Cerrado to over 2500 mm year^−1^ along the eastern Amazon boundary^16^.

Droughts in Amazonia and the adjacent Cerrado region are usually related to El Niño events and/or warmer-than-usual Tropical North Atlantic Sea Surface Temperatures. These oceanic temperature increases favor the occurrence of anomalous regional water deficits, warmer temperatures, and intense fire seasons^17–21^. This has been the case for the exceptional droughts in Amazonia in 2005^22^, 2010^23,24^, 2015–2016^25–27^, in the Pantanal 2019–2020^28,29^. These events are also occurring under a regime of regional warming and drying trends, more evident from the 80s on, particularly over Southeastern Amazon^2,27,30–38^. But water scarcity and higher temperatures can be limiting factors for soybean development, harvest, and production in the MATOPIBA. In the Cerrado region of Northeast Brazil, during El Niño years there is an increase of 20% in evapotranspiration together with irregular rainfall, leading to drought situations mainly in the states of Maranhão and Piaui and with rainfall below average precipitation in the rainy season in some municipalities of MATOPIBA^39^. El Nino is often associated with irregular or later-than-normal rains in Brazil’s Cerrado, including the main center-west grain belt, due to reduction in the number of rainy days^40^. The impact of La Niña on the growing season characteristics of Central Brazil was by delaying the onset of the rainy season and the growing season^41^. A reduction in soybean productivity was observed during El Niño in 2015/2016 (95.434.600 TM) as compared to 2014–2015 (96.243.300 TM)^42^. This region has experienced warming over the last three decades, and from 2003 to 2013 cropland agriculture more than doubled in area from 1.2 to 2.5 million ha, with 74% of new croplands sourced from previously intact Cerrado vegetation^43,44^.

Projected future deforestation scenarios over Amazonia show increases in the frequency of occurrence of longer dry seasons^7,42–48^. In addition, forest degradation has become a more important driver of carbon losses than deforestation^49^, including irrecoverable carbon stores within the Amazon basin^50^. Changes in MATOPIBA's fire regime resulting from rapid land expansion are still poorly understood, but it is suggested that climate is also playing a relevant role in this region^9,51^, which could put at risk both food production and EAC biome stability. We investigated this possibility, using 40 years of climate data to test for evidence of an intensification of hydro-climatic trends over the EAC. We show that this region is indeed suffering an intensification of combined drought-heat extremes particularly during recent decades, with the dry-to-wet season becoming warmer, drier, and longer, thus increasing climate and fire risks in the region.

## Results

### Compound changes in hydrological and climate variables

**Figure 1:**
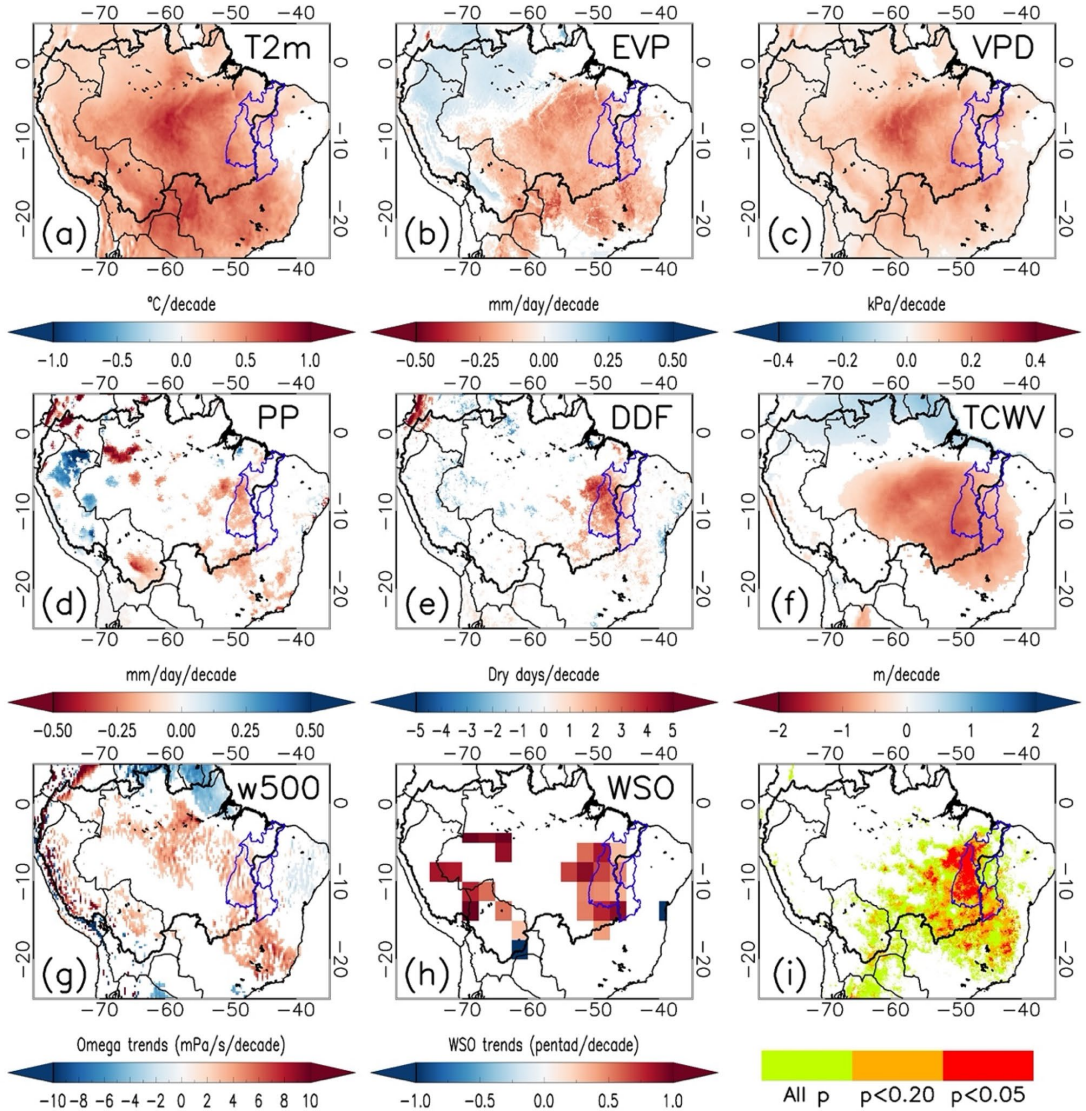
Spatial patterns of trends and compound changes. Trends (1981–2020) for the JASO seasonal period in air temperature T2m (**a**), actual total evapotranspiration EVP (**b**), vapor pressure deficit VPD (**c**), precipitation PP (**d**), frequency of dry days DDF (**e**), atmospheric water vapor content TCWV (**f**), omega at 500 hPa (**g**) and wet season onset WSO **(h)**. Values of trends given in units per decade. Only pixels statistically significant at the α = 0.05 level are displayed. Hydrological and climate changes are combined to display those pixels where positive trends in T2m, VPD and DDF, and negative trends in PP, EVP and TCWV are simultaneously observed (**i**). Compound trends are categorized into three levels: pixels without statistical significance (All p), pixels statistically significant at p < 0.2, and pixels statistically significant at p < 0.05. In all maps, the Amazon and MATOPIBA regions are marked by black and blue contour lines, respectively. Data visualisations produced using IDL v8 (Harris Geospatial Solutions, Inc).

Regions suffering a long-term warming and/or drying trend (1981–2020) are identified through the analysis of spatial patterns of these trends for different radiative, atmospheric, and hydrological variables (Fig. 1). EAC experienced a widespread and significant warming trend (0.38 ± 0.15 °C/decade, p < 0.05) during the dry-to-wet transition season July–October (JASO) over last four decades (Fig. 1a). The observed actual evapotranspiration (EVP) reduction (Fig. 1b) tends to elevate temperature, which increases sensible flux to offset the net downward radiative flux. Generally, an increase of surface net radiation and consequent increase of temperature lead to an increase of EVP if there is sufficient moisture in plants and soil. The widespread increase of vapor pressure deficit (VPD, Fig. 1c) is in line with the spatial pattern of warming observed in air temperature. This also agrees with global increases of VPD leading to reductions in vegetation growth^52^.

Overall, precipitation trends during the dry-to-wet transition season do not show a statistically significant widespread spatial pattern, but negative trends predominate over southern and southeastern Amazonia (Fig. 1d). A delayed wet season onset (WSO) is also noticed over eastern Amazonia (Fig. 1h), associated with an increase in atmospheric subsidence, as suggested by the positive trend in vertical velocity (omega) at 500 hPa over this region (Fig. 1g). Accordingly, EAC is characterized by a significant increase of the frequency of dry days (DDF, Fig. 1e). The observed DDF increases associated with increased subsidence over this region, are partially related to an intensification of the Hadley and Walker cells, and a higher frequency of winter weather types during September–October^53–55^. Moreover, the increase in DDF over this region is related to a warming of the northern tropical Atlantic Ocean and a weakening of moisture transport from the tropical Atlantic Ocean^31^. This is consistent with previous findings demonstrating the increased dry season length^2,53,55,56^, also observed through the delayed WSO (Fig. 1h). There is a reduction in the atmospheric water vapor content (TCWV) in eastern Amazonia (Fig. 1f), and a northwest to southeast gradient, wet over the north and dry over the south.

In water-limited areas such as the eastern Amazon, however, an increase of temperature is unlikely to increase EVP, especially in the dry and dry-to-wet seasons. Variation in water availability governs EVP in the seasonally dry tropical forests in the south and southeast Amazon, towards the transition with the Cerrado biome^24,57^. Dry-adapted plants can control stomata opening or shed their leaves in response to water deficits, but unadapted plants cannot. If the stomata are closed for too long, an increase in plant mortality by carbon starvation is expected. On the other hand, if plants are unable to avoid water loss, mortality is likely to increase because of cavitation^52^. This mechanism works well for tall Amazon trees in central Amazonia affected by drought, but Eastern Amazon trees maintain evapotranspiration during five-month dry periods by absorbing water from the soil to depths of more than 8 m^58^. The Cerrado has their own mechanisms to limit transpiration while still surviving the dry season, including deciduousness, leaf anatomical and morphological characteristics, canopy structure, and soil hydraulic mechanisms^59^.

All these processes are fundamentally linked to canopy–atmosphere coupling, with complex interactions between climate, plant phenology and soil hydraulic mechanisms.

**Figure 2:**
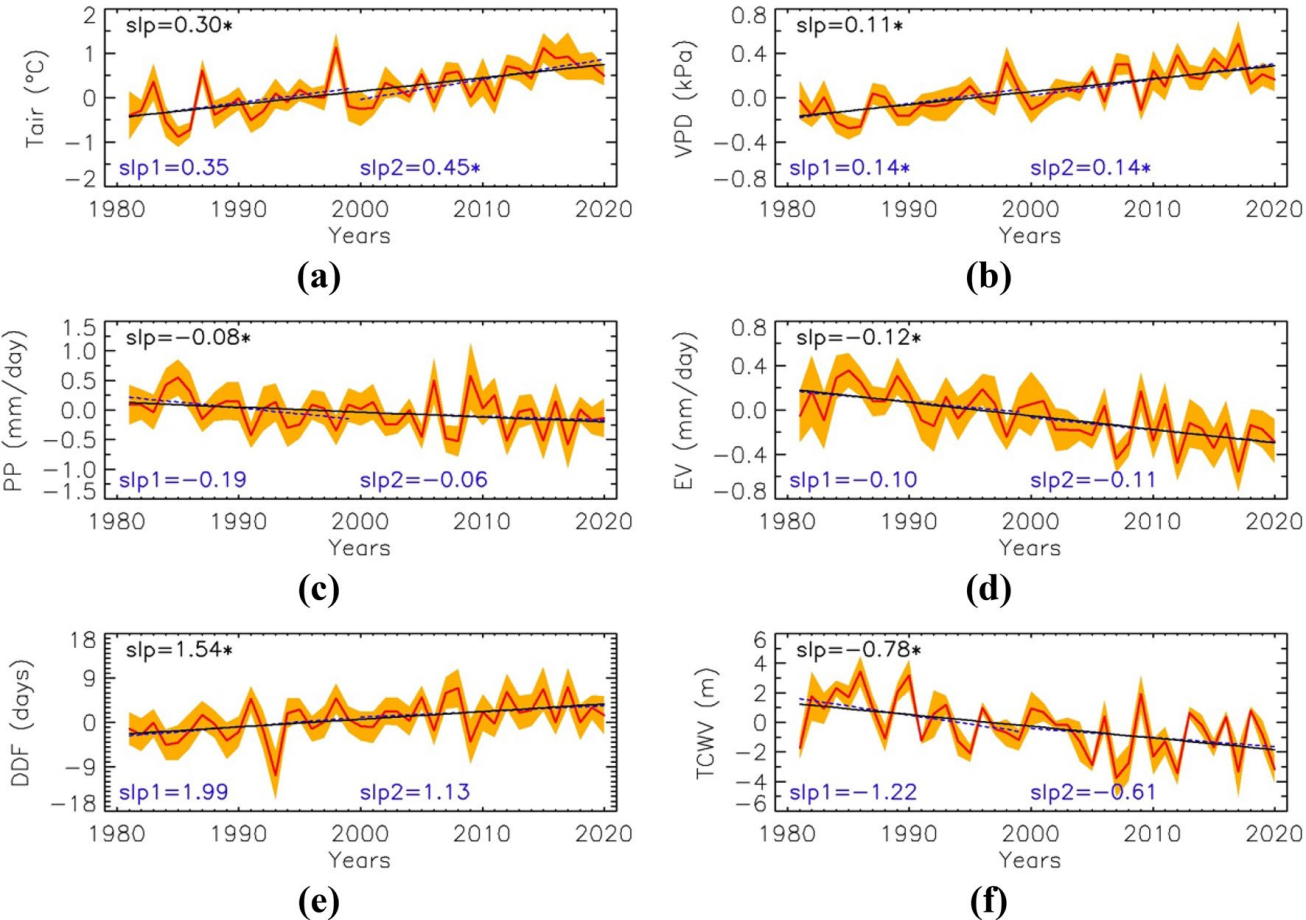
Trends of atmospheric and hydrological variables over the MATOPIBA region. Time series (1981–2020) of anomalies in air temperature (Tair) (**a**), vapor pressure deficit (VPD) (**b**), precipitation (PP) (**c**), actual evapotranspiration (EV) (**d**), dry-day frequency (DDF) (**e**), and total atmospheric column water vapor (TCWV) (**f**). Slope of the linear trend for the periods 1981–2020 (slp, continuous line), 1981–1999 (slp1, dashed line) and 2000–2020 (slp2, dashed line) are also given. Values statistically significant at p < 0.05 marked with asterisks.

The analysis of long-term trends (Fig. 1a–h) evidenced that some of the hydrological and climate changes are already widespread in EAC, whereas other changes are focused on southern/southeastern Amazonia or even finer regional scales. By combining changes of all the variables into a single compound indicator (Fig. 1i), we show that EAC is suffering a combined dry and warming trend. The EAC sensitive region is mainly composed of Cerrado and encompasses roughly the MATOPIBA region (Extended Data Fig. 1). Therefore, the MATOPIBA region shows the strongest heating and drying trends observed across the whole of the Amazon and Tocantins basins and Cerrado biome (Fig. 2). This agrees with the fire distribution focused across the southern boundary of the Amazon basin and in the EAC during the May–August dry season because the disturbed forests are more prone to burning in the dry-to-wet transition season than in the wet and dry seasons^60^.

### Regional scale trends over the MATOPIBA

The EAC, including MATOPIBA has already been changing towards a drier and warmer climate (Fig. 2). The warming trend (Fig. 2a), is 0.3 °C/decade (p < 0.05); the total change from 1981 to 2020, 1.2 °C. For the post-2000 period it increases to 0.45 °C/decade (p < 0.05), giving an increase of almost 1 °C over the last two decades. The highest temperature anomalies (1.1 °C) are observed in 1998 and 2015 (strong ENs), and 2017 (0.9 °C). However, the MATOPIBA reached higher values of monthly-mean temperature anomalies, with a record 2.9 °C in December 2015 (Extended Data Fig. 2). Rainfall trends show systematic reductions (− 0.08 mm/day per decade, p < 0.05) and an increase in the DDF (+ 1.5 days per decade, p < 0.05) (Fig. 2b,c). The trend is slightly greater (around + 2 days per decade) during the first half of the period (1981–1999), but this is not statistically significant.

These decreases in rainfall since 1980 are related to positive changes in the vertical velocity omega (Fig. 1g), indicating increasing subsidence (less convective activity, thus dryness and warming) in the EAC region. This change since the late 90s has been identified in previous studies as part of changes in regional atmospheric circulation in JASO^30^. Warming and drying (temperature, precipitation and DDF) are stronger over Tocantins state (Extended Data Fig. 3) than anywhere else throughout the MATOPIBA region (Fig. 1i).

### Drought and land cover changes

Drought indices evidence a drying trend over EAC for the period 1981–2020 (Extended Data Fig. 4). There is a strong interannual change in SPI, SPEI and scPDSI, with consistently negative trends in all indices, consistent with the decreasing rainfall. SPI shows the smallest negative trend (p < 0.1). SPEI and scPDSI indices, which also include the impact of evapotranspiration, show a strong negative trend (p < 0.0001). While SPI and SPEI show the largest negative values in 2015 and 2017, scPDSI provides the largest negative value in 2016, probably because of the different response to soil moisture memory between indices. scPDSI also evidences a strong negative trend from year 2000. SPEI and scPDSI agree on the negative index value in 2020. In sum, all of them show a significant drying trend during the last four decades.

**Figure 3:**
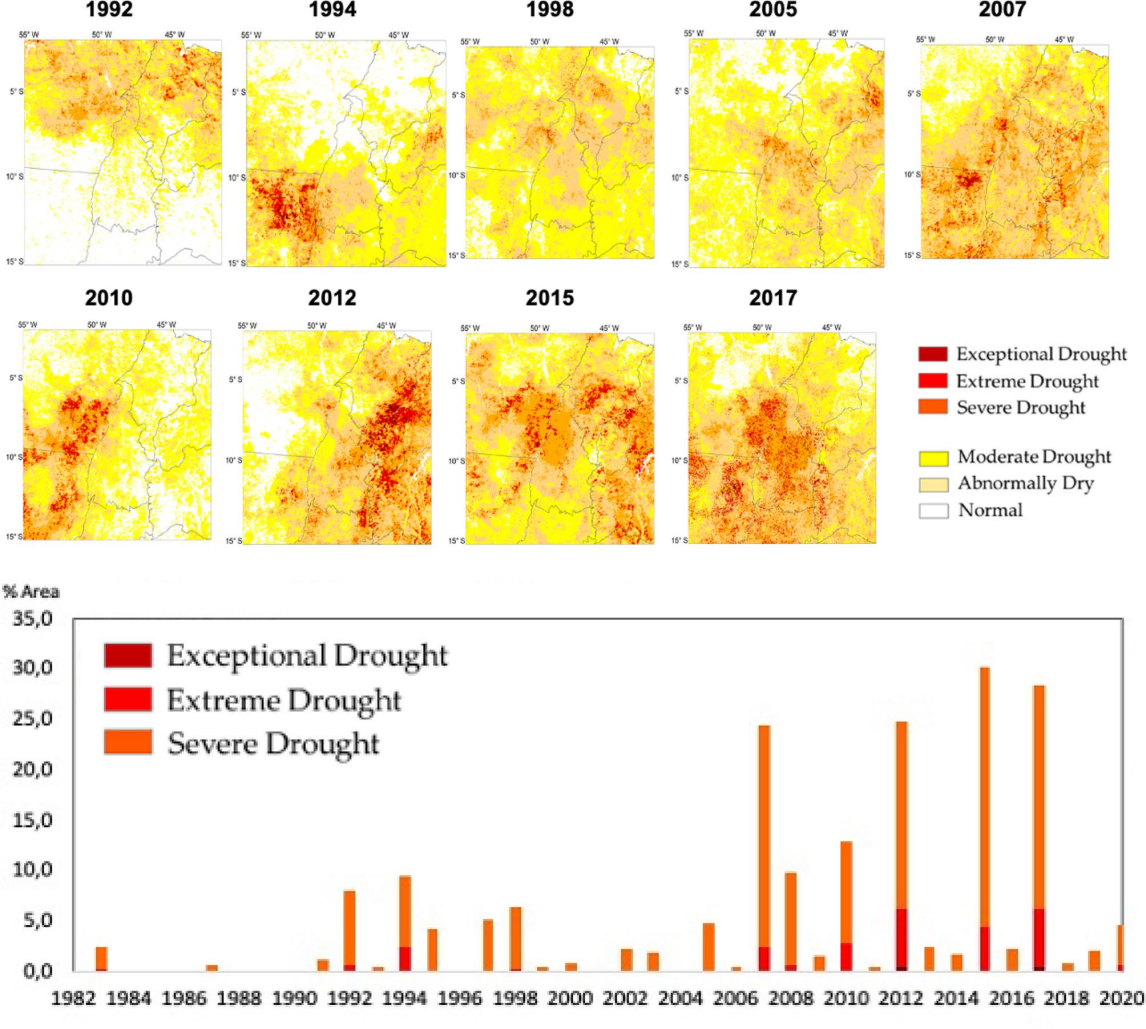
Evolution of drought area in the MATOPIBA regions. Time series of percentage of area affected by severe to exceptional drought from 1982 to 2020 using Integrated Drought Index (IDI). Spatial patterns of IDI are also shown for exceptional years. Maps in the upper level produced by using ArcMap 10.6; www.esri.com/en-us/home (https://www.esri.com/en-us/home).

The exposure of affected areas to drought and its intensity can be assessed by the Integrated Drought Index (IDI), which combines the lack of precipitation and the vegetation response to water stress (Fig. 3): 1992, 1994, 1998, 2005, 2007, 2010, 2012, 2015 and 2017 were years when the area affected by severe to exceptional drought increased. Some of these years are recognized as strong El Niño years (1998, 2015) or having an anomalously warm Tropical North Atlantic (2005, 2010). Note that the area impacted by drought was at least as large as that in other years (e.g., 2007, 2012, 2017). This suggests that the impacts of the exceptional droughts of 2005 and 2010 may be surpassed by the exacerbated dry condition already established in the region in recent years, and not necessarily attributable to El Niño or warm Tropical North Atlantic conditions. Consequently, during the period 2007–2020, more than 25% of the MATOPIBA region was affected by severe to exceptional drought in four years (2007, 2012, 2015 and 2017) (Fig. 3).

This trend towards an increase in drought frequency also occurs in a scenario of land cover changes in the EAC, characterized by conversions to pastures and croplands since the mid 1980s^61^. Global land cover satellite MODIS MCD12C1 products from 2001 to 2019 show that area of forest has diminished gradually at a rate of 2.8% (p < 0.01) per decade in the last 20 years (Extended Data Figs. 5, 6). In contrast, the combination of savanna, grasslands, and croplands increased significantly. Between 2002 and 2011, deforestation rates in the Cerrado (1% per year) were 2.5 times higher than in the Amazon basin (EMBRAPA-www.embrapa.br). This overall drying and warming trend over MATOPIBA may depend on land cover (Extended Data Fig. 7), with trends in air temperature and evaporation over forests (EBF class) resembling trends over areas converted from forest to savannah and grasslands (Extended Data Fig. 8). The warming trend, however, is not necessarily in line with precipitation deficits (trends in precipitation are not statistically significant, except for the savannah class and the period 1981–2020). However, because of the wide magnitude of trends (error bars in Extended Data Fig. 7), our analysis do not allow to conclude that these trends are statistically different across land covers.

Long-term trends of air temperature and evaporation variables documented in this study show that dry conditions have intensified during the last two decades, suggesting increasing water stress on vegetation. Dry and dry-to-wet seasons in eastern Amazonia are becoming longer, warmer and dryer. While deforestation in eastern Amazonia along the deforestation arc has increased in recent years, intensified fire seasons have occurred when drought conditions affect this region, and the impacts of compounded drought-heat events in eastern Amazonia extend to the Cerrado vegetation along the Tocantins River basin.

Analysis of evaporation and dry-day frequency agrees with previous studies that show a relationship between a delay of the wet season onset and area deforested and could further reduce evaporation and exacerbate the dryness over Amazonia^5,60^.

### Socioeconomic and ecological implications

In 2015 the Brazilian government declared MATOPIBA to be the country’s “last agricultural frontier”^62^. The expansion of the MATOPIBA agricultural frontier, driven by agribusiness, is imposing a new functional reorganization on the use and occupation of the territory. In recent years this region has sacrificed a large part of native vegetation for soybean and cattle production. The expansion of agribusiness, and especially of soybean monocultures, began in this region since the 1980s, and accelerated in the early 2000s^60^. In the last two decades, the area cultivated with soybeans in South America more than doubled, with most of the soybean-driven deforestation observed in the Cerrado^63^ (Extended Data Fig. 9). In 2012, there were about 2.5 million hectares cultivated with soy in the MATOPIBA, producing more than 7 million tons and generating revenues exceeding R$5.5 billion (USD 1.05 billon).

Currently, almost a quarter of the Cerrado’s soybean area is in MATOPIBA^64^, where most of the agricultural expansion in occurred over native vegetation—68% (780,000 hectares) between 2000 and 2007, and 62% (1.3 million hectares) in the following period, between 2007 and 2014, especially in Maranhão and Piaui, the current agricultural frontier of the Cerrado.

## Discussion

In eastern Amazonia, where the rates of deforestation are higher, our study showed a tendency toward longer, warmer, and drier dry-to-wet seasons (JASO), suggesting late onsets of the rainy seasons. These conditions favor higher risk of water stress and seem to extend outside the eastern Amazon to the transition with the Cerrado region, and to the MATOPIBA, where soybeans are growing. Dangerous climate trends detected in the EAC may put at risk agricultural production and forest stability and the Cerrado’s natural vegetation processes and consequent ecosystem services, which may affect agriculture here. MATOPIBA partially emerged and consolidated itself as a program supported by the Brazilian government for the expansion of agribusiness. This represents a shift away from Amazonia policies, in response to strong opposition to deforestation there. However, environmental change processes, driven by the socioeconomic growth in the MATOPIBA region will in turn be strongly affected by changing climatic characteristics, and throughout the region, the gradual increase in annual temperature and water deficit are consistent with a longer and warmer dry season, with a high frequency of very warm days. Since soybean productivity is affected by rainfall deficits during the wet season from March to April (Extended Data Fig. 10), this suggests that the continuation of soybean expansion over areas with a clear drying trend can reduce productivity, putting at risk food security and the Brazilian economy.

We conclude that a key region responsible for agricultural production in Brazil is at increased risk of climate-driven impacts. This can affect the productivity and even restrict the suitability of this area for further expansion. Changes already observed are critical and may put food security at risk. Soybeans are the major food animal feed commodity produced in the Brazilian Cerrado, and heat and drought conditions in this region may be affected by the increasing water deficit conditions over the last 20 years. The consequences of climate change and deforestation in both Amazonia and Cerrado could bring this agribusiness boom to an end.

## Methods

### Study area and land cover

The study area includes the Amazon and Tocantins River Basins, including the MATOPIBA region (Extended Data Fig. 1). Land cover identification and change was performed through the MODIS Land Cover product (MCD12C1, version 6), which provides yearly land cover classes on a grid of resolution of 0.05°^65^. The main land cover over the study area is evergreen (broadleaved) forest, except for eastern Amazonia (approximately the Tocantins River Basin and Brazilian states of Maranhão, Tocantins and Goiás), characterized by the presence of the Cerrado biome with a mixture of forest, savanna-like vegetation, and croplands (Extended Data Fig. 1).

### Precipitation and dry-day frequency

Precipitation data comes from the Climate Hazards Group InfraRed Precipitation CHIRPS^66^ (downloaded at https://data.chc.ucsb.edu/products/CHIRPS-2.0/). CHIRPS combines satellite and rain gauge data, as well as global cold cloud duration (CCD), as a thermal infrared method to estimate the global precipitation. Precipitation estimated by the global CCD is then calibrated using the TRMM-3B42 V7 product. The CHIRPS data set has a horizontal resolution of 0.05° × 0.05° and extends from January 1981 to the present. This dataset has been proved to be a valid precipitation product over Amazonia^67^. We used monthly values of CHIRPS precipitation data for the analysis of temporal series and trends.

Dry-Day Frequency (DDF) was calculated from CHIRPS daily data^31^, by evaluating in each grid box the evolution of the number of days per JASO season during the period 1981–2020, with rainfall below 1 mm/day. The Kendall trend test is then applied to gridded DDF time series to analyze its evolution (Figs. 1e, 2e).

### Climate data

Climate data was extracted from the Climate Data Store (CDS) of the Copernicus Climate Change Service (C3S) (https://cds.climate.copernicus.eu/). Air temperature at 2 m, total atmospheric water vapor content (TCWV), vertical velocity (omega) at 500 hPa, specific humidity at 1000 hPa, and surface pressure were generated from the European Centre for Medium-Range Weather Forecasts ECMWF Reanalysis Version 5 (ERA5)^68^. ERA5 provides hourly estimates of several atmospheric, land and oceanic climate variables. The data have a horizontal resolution of 30 km with 137 vertical levels from the surface up to 80 km and are available from 1979 to the present. Fields of air temperature, specific humidity and surface pressure were used for the computation of the vapor pressure deficit (VPD) as the difference between saturation vapor pressure (es) and actual vapor pressure (ea)^69^.

Air temperature at 2 m and total evaporation were obtained from the land component of the ERA5 reanalysis (ERA5-Land)^68^. ERA5-Land includes a series of improvements including an enhanced grid resolution of 1° × 1° making it more accurate for all types of land applications.

### Wet season onset

The wet season onset (WSO) was calculated using the outgoing longwave radiation (OLR) at resolution 2.5° × 2.5° from NOAA (psl.noaa.gov/data/gridded/data.interpOLR.html#detail)^70^ as a proxy for deep convection in the tropical region. The WSO was calculated based on OLR pentad averages at each grid point^71^. WSO was identified as the pentad after which OLR values are less than 240 W/m^2^ in 8 of 12 consecutive pentads, and just after having been exceeded in an equal number of pentads.

### Meteorological-based drought indicators: scPDSI, SPI and SPEI

The self-calibrated Palmer Drought Severity Index (scPDSI) automatically calibrates the behavior of the PDSI index (Palmer 1965) at any location by replacing empirical constants in its computation with dynamically calculated values^72^. The scPDSI product was computed from the CRU rainfall dataset. The Standardized Precipitation Index (SPI) drought indicator^68^, measures precipitation anomalies at a given location, with respect to observed precipitation over a specified time intervals of (e.g. 1, 3, 12, 48 months), with the long-term historic rainfall for a given interval. SPI is calculated using the gridded rainfall data from CHIRPS^66^. The Standardized Precipitation-Evapotranspiration Index SPEI is a multiscale drought index based on climatic data, which can be used for determining the onset, duration, and magnitude of drought conditions with respect to normal conditions for a variety of natural and managed systems such as crops, ecosystems, rivers, water resources, etc.^72^. To calculate SPEI we use monthly precipitation from CRU. Both SPI and SPEI were calculated for the box in the eastern Amazon-Cerrado transition zone for the JASO season.

### Integrated drought index (IDI)

In this study, we used the integrated drought index IDI^73^, which combines a meteorologically-based drought index and a remote sensing-based index, to assess the drought events from 2001 to 2019 over the eastern Amazonia-Cerrado and the MATOPIBA transition region, calculated for the JASO season.

### Soybean area

Soybean area, classified from remote sensing satellite data (Extended Data Fig. 9), was extracted from datasets at https://glad.umd.edu/projects/commodity-crop-mapping-and-monitoring-south-america. Annual maps of soy cover across South America were downloaded in GeoTif format^63^ and resized over the MATOPIBA region. Soybean yield (kg/ha) over the region (Extended Data Fig. 10) was obtained from IBGE statistics available at https://sidra.ibge.gov.br/pesquisa/pam/tabelas (accessed on May 10 2021).

We observed a weak but statistically significant correlation (Kendall’s tau of 0.33, p < 0.005) between annual soybean production in MATOPIBA and March–April precipitation anomalies after trend removal (Extended Data Fig. 10).

### Seasonal anomalies and trends

Anomalies and trends of the different variables were obtained for the July to October (JASO) season for the period 1981–2020. JASO This was considered because it includes the dry season and the season of transition from dry to wet. It is also the Cerrado’s dry season, with the highest fire incidence in this biome^9^. The reference period for computing anomalies was 1980–2010. Although some datasets extend the study period back to the 1950, our study was limited to 1981–2020 because the CHIRPS precipitation dataset is only available from 1981 on; also, reanalysis data is also more reliable from the 80s on. In the case of variables extracted from satellite products (MODIS Land Cover, Soybean classification), the study period was limited to 2001–2020.

The Mann–Kendall test and Sen’s slope estimator were used for trend detection^74^. Linear regression was used to plot linear trends onto the graphs of temporal series.

## Supplementary Information

Supplementary Figures.
