# Supplementary material for: Increased climate pressure on the agricultural frontier in the Eastern Amazonia–Cerrado transition zone
Source: Sci Rep. 2022 Jan 10;12:457. doi: 10.1038/s41598-021-04241-4 (PMC8748735; doi:10.1038/s41598-021-04241-4)
Supplement: Supplementary file 1 — Supplementary Figures. [file 41598_2021_4241_MOESM1_ESM.pdf]

Increased climate pressure on the agricultural frontier in the Eastern Amazonia-Cerrado transition zone

José A. Marengo[1], Juan C. Jimenez[2], Jhan-Carlo Espinoza[3], Ana Paula Cunha [1], and Luiz E. O. Aragão [4]

[1]CEMADEN, São Jose dos Campos - SP, Brazil

[2]GCU/IPL, Univerdity of Valencia, C/Catedratico Jose Beltran, 46980, Paterna (Valencia), Spain

[3]Université Grenoble Alpes, IRD, CNRS, G-INP, IGE (UMR 5001), Grenoble, France

[4] Remote Sensing Division, National Institute for Space Research INPE, Av. dos Astronautas, 1.758, 12227-010 Sao José dos Campos, Brasil.

Corresponding author information:

Jose A. Marengo, CEMADEN, jose.marengo@cemaden.gov.br

Extended data figures

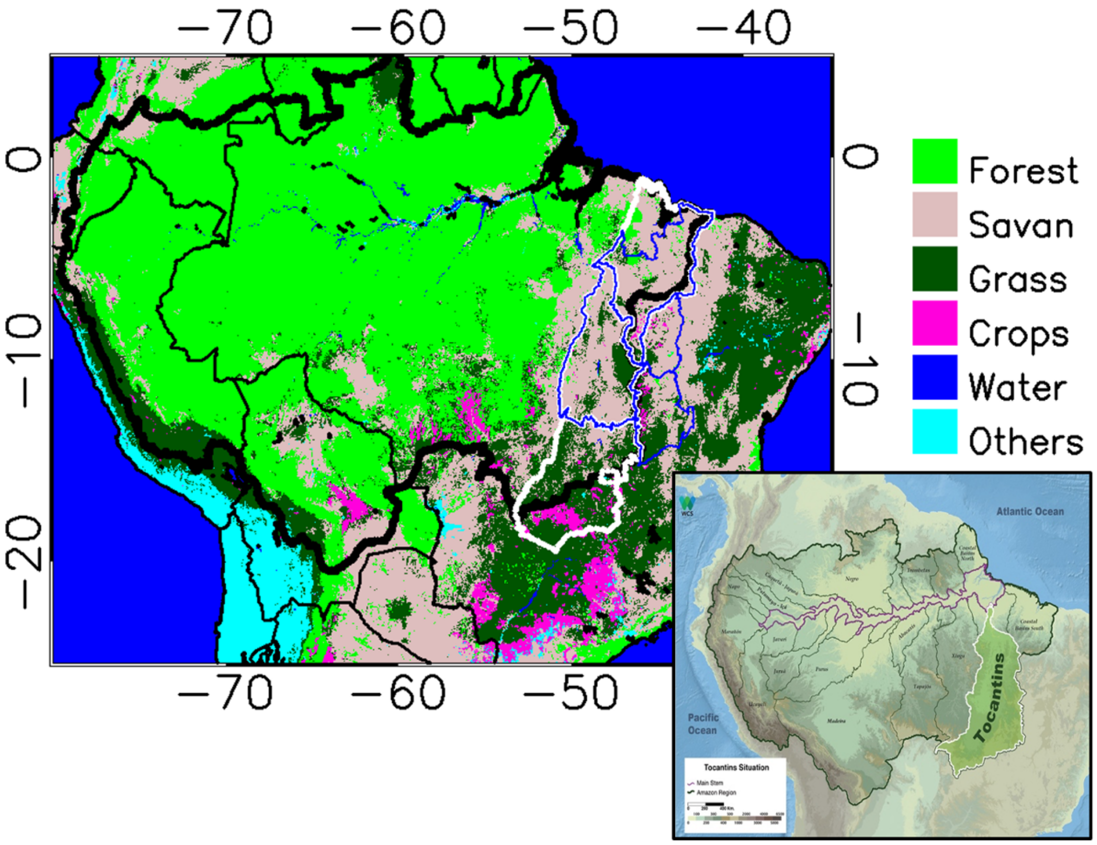

Extended Data Fig. 1. Study area: Amazon and Tocantins River Basins. Main land covers over the study area extracted from the MODIS Land Cover product MCD12C1v6 (year 2019) are included for reference. Black contour delimits the Amazon and Tocantins River Basins; white contour shows the states of Maranhao, Tocantins and Goias States; blue contour marks the MATOPIBA region. Data visualisations produced using IDL v8 (Harris Geospatial Solutions, Inc). Map on the bottom right corner extracted from aguasamazonicas.org.

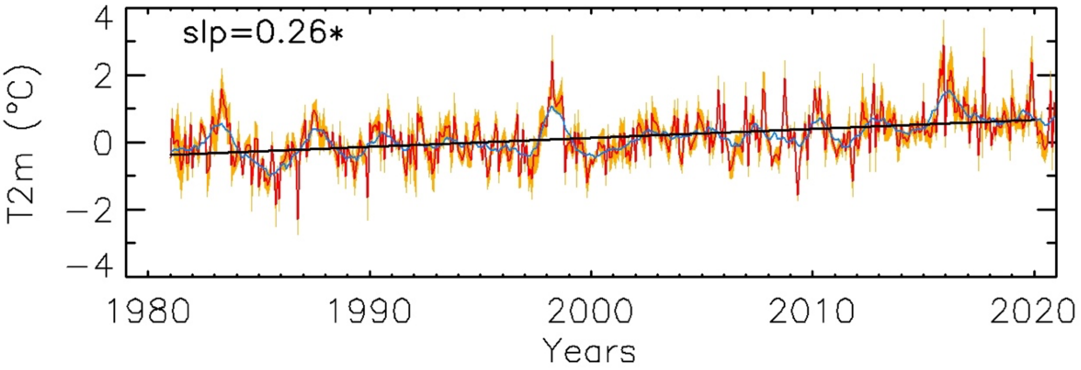

Extended Data Fig. 2. Trends in air temperature over the MATOPIBA region. Temporal series (1981-2020) of monthly anomalies in air temperature. The slope of the linear trend (slp, continuous line) is also given. Values statistically significant at $p<0.05$ are marked with an asterisk. The blue line represents a moving average filter of 12 months.

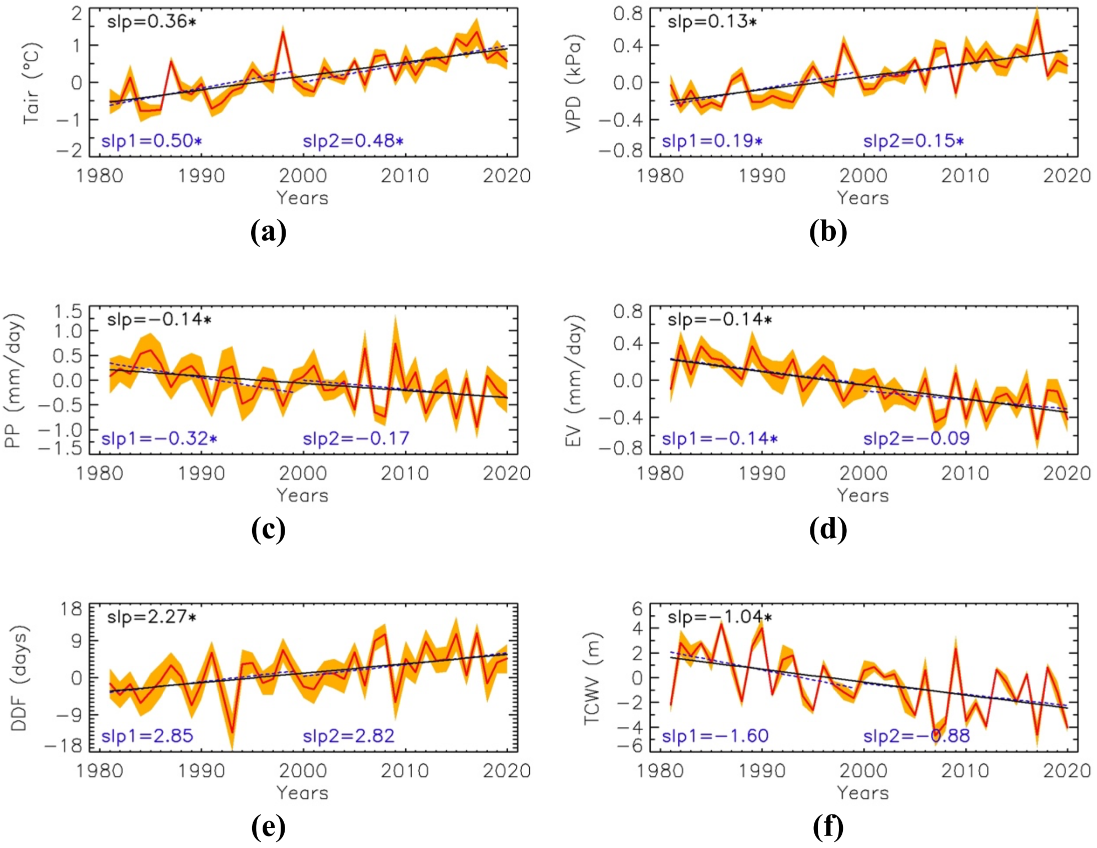

Extended Data Fig. 3. Trends of atmospheric and hydrological variables over Tocantins state. Temporal series (1981-2020) of anomalies in air temperature (Tair) **(a)**, vapor pressure deficit (VDP) **(b)**, precipitation (PP) **(c)**, actual evapotranspiration (EV) **(d)**, dry-day frequency (DDF) **(e)**, and total atmospheric water vapor column (TCWV) **(f)**. The slope of the linear trend for the period 1981-2020 (slp, continuous line), period 1981-1999 (slp1, dashed line) and period 2000-2020 (slp2, dashed line) are also given. Values statistically significant at $p<0.05$ are marked with an asterisk.

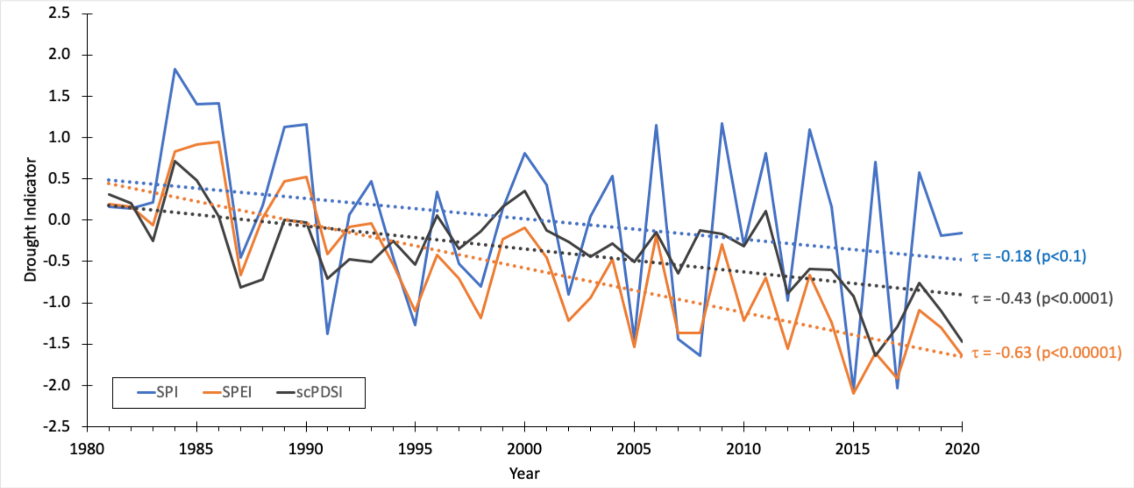

Extended Data Fig. 4. Interannual variability of drought indices over the MATOPIBA region. Times series of seasonal (JASO) SPI, SPEI, and scPDSI drought indices. SPI was computed from CHIRPS rainfall data, while SPEI and scPDSI use CRU rainfall data. SPI and SPEI are provided at a time interval of 4 months.

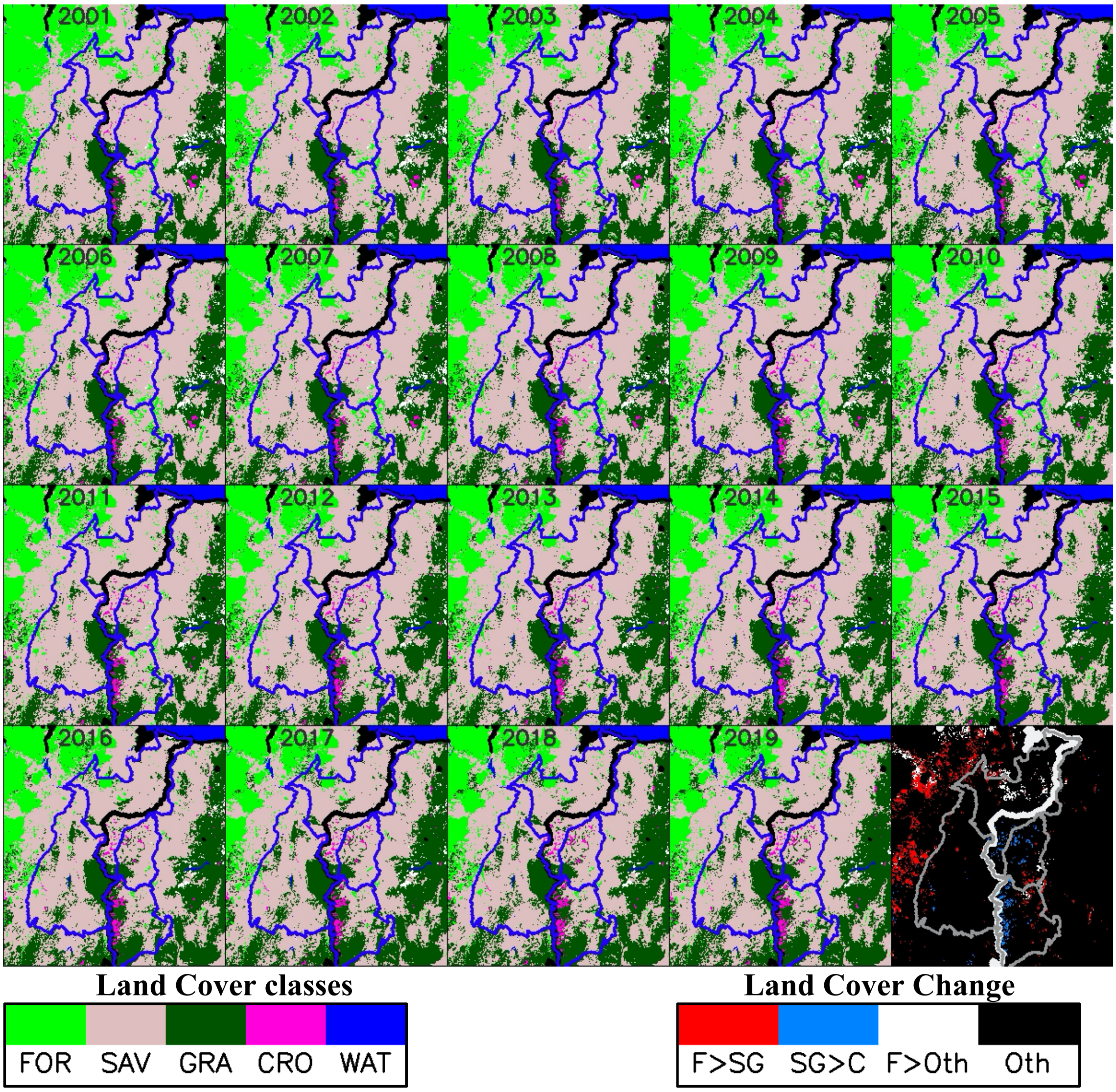

Extended Data Fig. 5. Main land covers (MODIS MCD12C1v6) for the eastern Amazonia-Cerrado transition zone from 2001 to 2019. Last panel shows areas with changes from forest to savanna+grassland (F>SG) in red color, forest to other land covers (F>Oth) in white color, and savanna+grassland to crops (SG>C) in blue color. Other changes are coloured in black. Data visualisations produced using IDL v8 (Harris Geospatial Solutions, Inc).

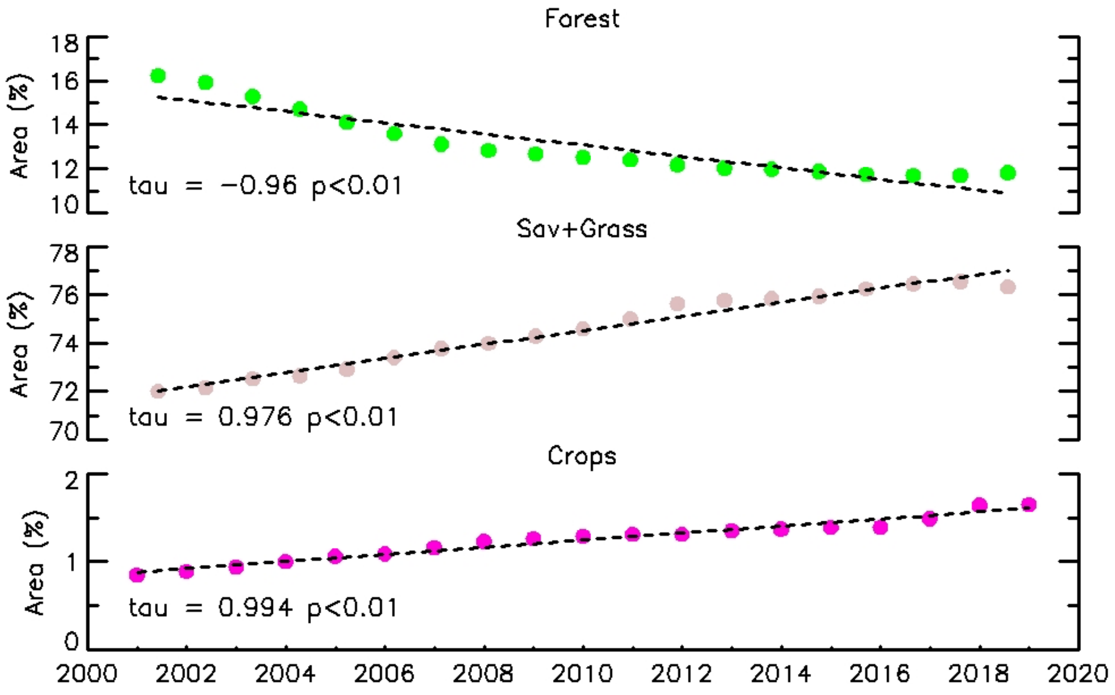

Extended Data Fig. 6. Time series of land cover area from 2001 to 2019 using global MODIS Land Cover product (MCD12C3). Only forest, combined savanna and grasslands, and croplands are presented. Kendall's tau and p-value are also given.

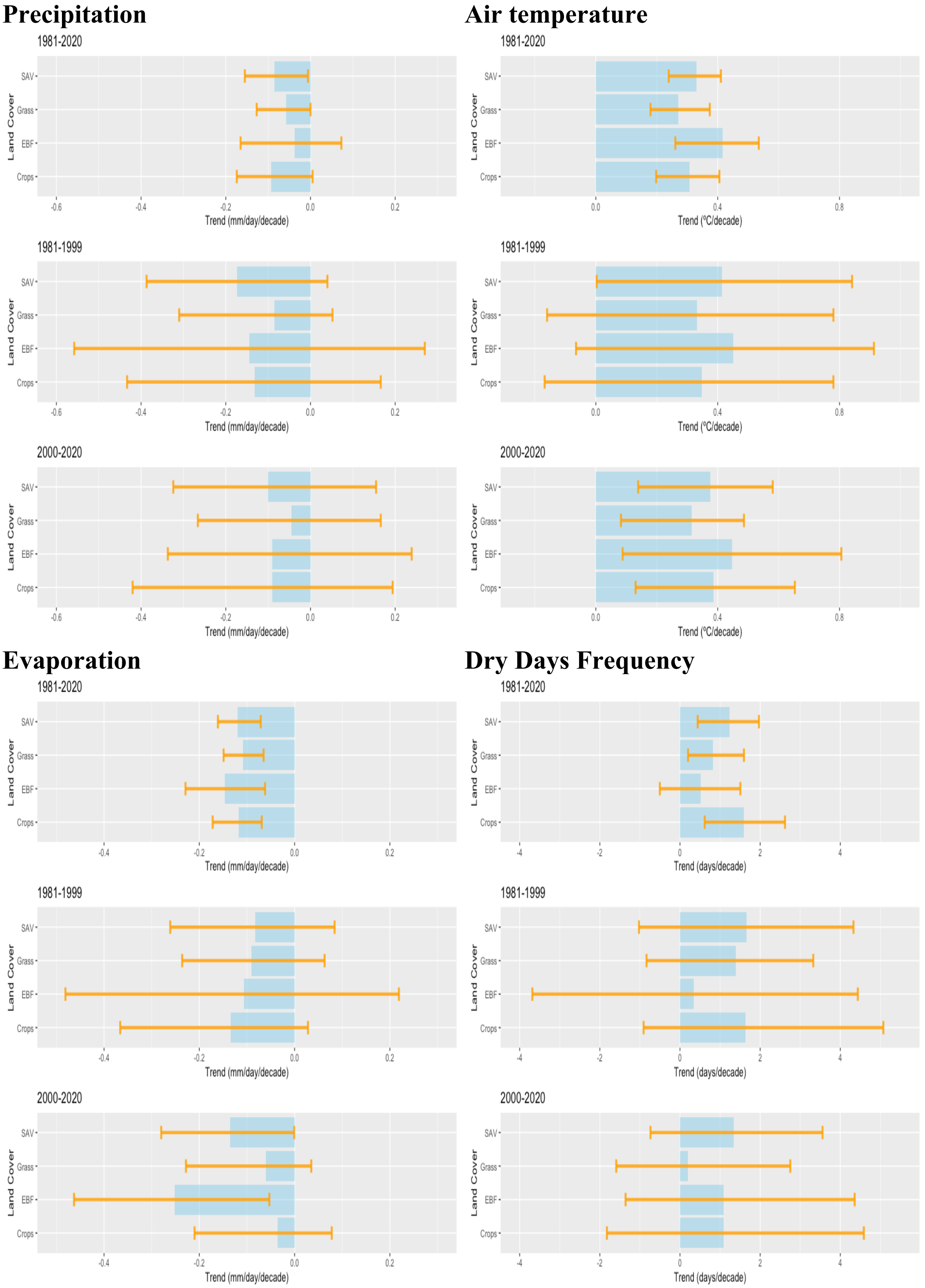

Extended Data Fig. 7. Trends of atmospheric and hydrological variables over MATOPIBA region for different land cover classes (see Extended Data Fig. 5). Trends in precipitation (mm·day^{-1}·decade^{-1}), air temperature (ºC·decade^{-1}), evaporation (mm·day^{-1}·decade^{-1}), and dry-day frequency (days per decade) for land cover classes of evergreen broadleaf forests (EBF), savannah (SAV), croplands (CROPS), and grasslands (GRASS). Trends are provided for periods 1981-2020, 1991-1999, and 2000-2020. The

mean value of the slope is represented by blue bars, whereas the lower and upper limits of the slope at the 95% confidence interval are represented by the orange error bars. Trends were calculated using the Sen's slope estimator.

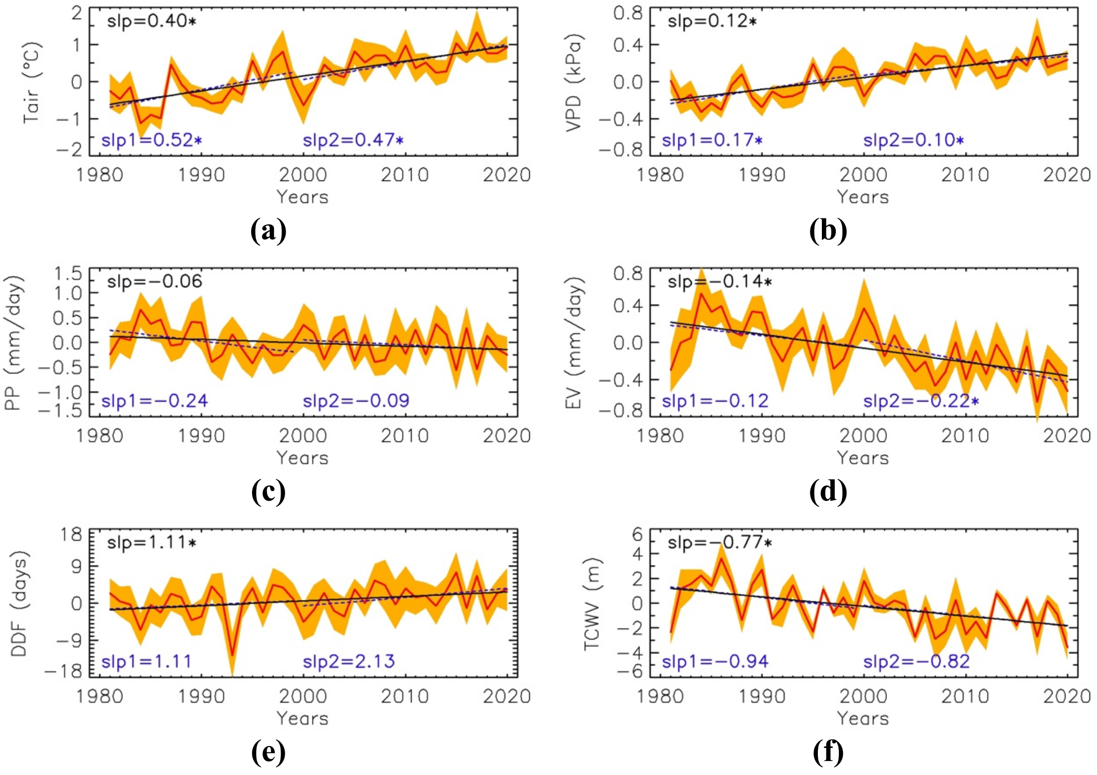

Extended Data Fig. 8. Trends of atmospheric and hydrological variables over MATOPIBA region for only those areas affected by conversion from forest to savanna and grassland (see Extended Data Fig. 5). Temporal series (1981-2020) of anomalies in air temperature (Tair), vapor pressure deficit (VDP), precipitation (PP), actual evapotranspiration (EV), dry-day frequency (DDF), self-calibrated Palmer Drought Severity Index (scPDSI), total atmospheric column water vapor (TCWV), and omega. Slope of linear trend line for the periods 1981-2020 (slp, continuous line), 1981-1999 (slp1, dashed line) and 2000-2020 (slp2, dashed line) are also given. Values statistically significant at $p<0.05$ marked with asterisks.

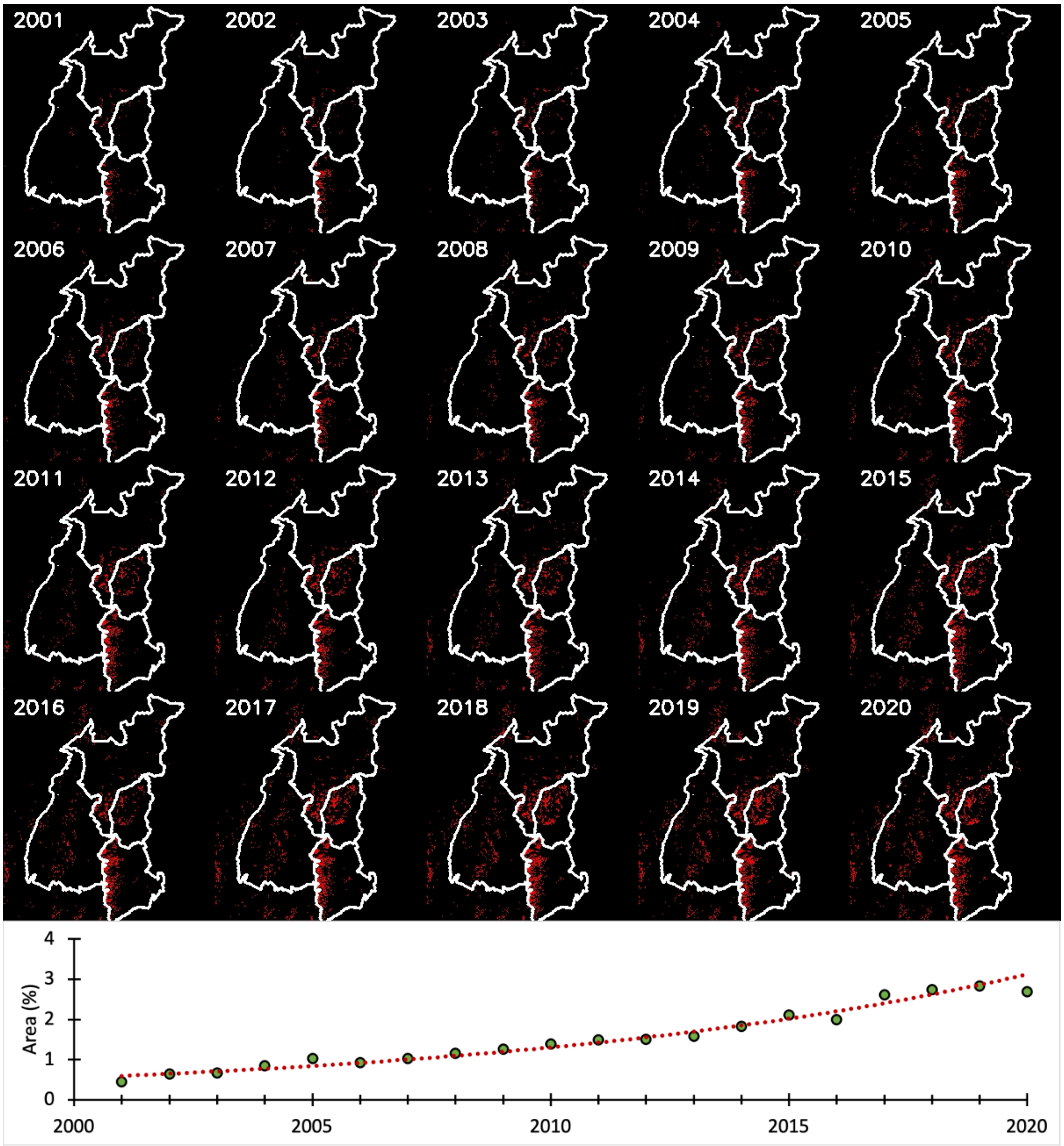

Extended Data Fig. 9. Annual soybean expansion in the MATOPIBA region. Satellite-based soybean classification (red areas) from 2001 to 2020. Relative abundance of soybean (in percentage) plotted below; dotted line represents an exponential fit. Data visualisations produced using IDL v8 (Harris Geospatial Solutions, Inc).

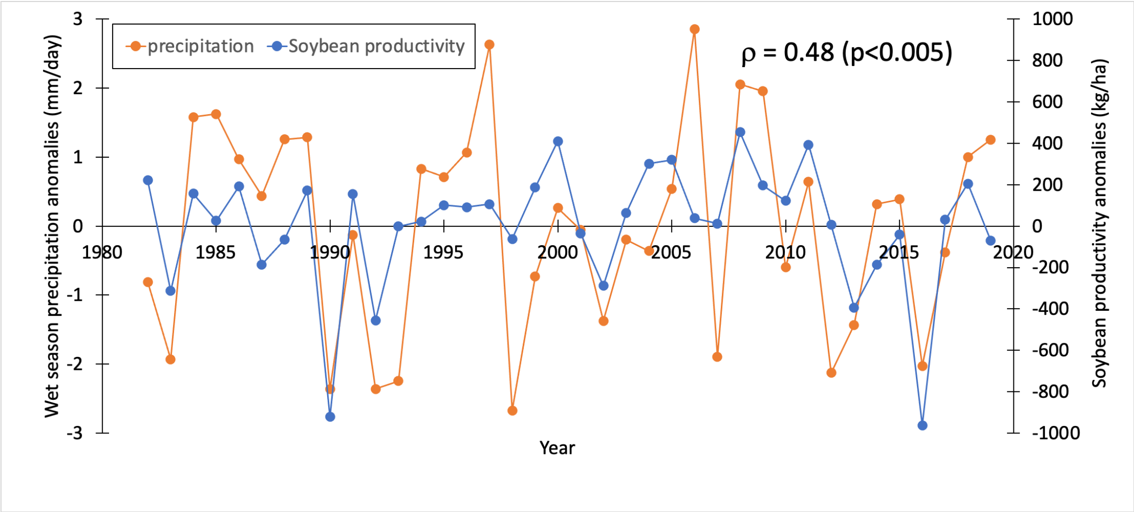

Extended Data Fig. 10. Correlation between annual soybean productivity anomalies and wet season (March-April) rainfall anomalies after trend removal. Spearman's rank correlation rho (ρ) and p-value are included.
